# Quantitative proteomic analyses reveal that energy metabolism and protein biosynthesis reinitiation are responsible for the initiation of bolting induced by high temperature in lettuce (*Lactuca sativa* L.)

**DOI:** 10.1186/s12864-021-07664-5

**Published:** 2021-06-09

**Authors:** Jing-hong Hao, He-Nan Su, Li-li Zhang, Chao-jie Liu, Ying-yan Han, Xiao-xiao Qin, Shuang-xi Fan

**Affiliations:** 1 Beijing Key Laboratory of New Technology in Agricultural Application, National Demonstration Center for Experimental Plant Production Education, Plant Science and Technology College, Beijing University of Agriculture, No. 7 Beinong Road, Huilongguan town, Changping district, Beijing, 102206 China; 2 Yulin Academy of Agricultural Sciences, Yulin, 719000 China

**Keywords:** Lettuce, High temperature, Bolting initiation, Proteome, iTRAQ

## Abstract

**Background:**

Lettuce (*Lactuca sativa* L.), one of the most economically important leaf vegetables, exhibits early bolting under high-temperature conditions. Early bolting leads to loss of commodity value and edibility, leading to considerable loss and waste of resources. However, the initiation and molecular mechanism underlying early bolting induced by high temperature remain largely elusive.

**Results:**

In order to better understand this phenomenon, we defined the lettuce bolting starting period, and the high temperature (33 °C) and controlled temperature (20 °C) induced bolting starting phase of proteomics is analyzed, based on the iTRAQ-based proteomics, phenotypic measurement, and biological validation by RT-qPCR. Morphological and microscopic observation showed that the initiation of bolting occurred 8 days after high-temperature treatment. Fructose accumulated rapidly after high-temperature treatment. During initiation of bolting, of the 3305 identified proteins, a total of 93 proteins exhibited differential abundances, 38 of which were upregulated and 55 downregulated. Approximately 38% of the proteins were involved in metabolic pathways and were clustered mainly in energy metabolism and protein synthesis. Furthermore, some proteins involved in sugar synthesis were differentially expressed and were also associated with energy production.

**Conclusions:**

This report is the first to report on the metabolic changes involved in the initiation of bolting in lettuce. Our study suggested that energy metabolism and ribosomal proteins are pivotal components during initiation of bolting. This study could provide a potential regulatory mechanism for the initiation of early bolting by high temperature, which could have applications in the manipulation of lettuce for breeding.

**Supplementary Information:**

The online version contains supplementary material available at 10.1186/s12864-021-07664-5.

## Background

In the life cycle of flowering plants, bolting is a floral transition involving an important developmental phase switch from vegetative to reproductive growth [1]. After bolting, the floral stems rapidly elongate, and the flower buds begin to differentiate. Early bolting of leafy and root vegetables leads to poor quality of plants and fields, resulting in loss of edibility and commercial value; therefore, it is very important to prevent bolting.

In the progress of bolting, the shoot apical meristem (SAM) elongates and changes into the inflorescence meristem (IM). This phenotype is regulated by endogenous and environmental factors, including vernalization, gibberellin (GA), photoperiod, ambient temperature, and autonomous and age-related pathways [2, 3]. In *Arabidopsis thaliana*, many genes have been shown to participate in the floral transition. Among the three transcription factors, *FLOWERING LOCUS T (FT), SUPPRESSOR OF OVEREXPRESSION OF CONSTANS1 (SOC1)* and *LEAFY (LFY)* act as the main integrators that control the eventual flowering time [4, 5].

Previous research on bolting has mostly been conducted on “vernalization”-type plants such as cabbage [6, 7], onion [8], spring cabbage [9, 10], and radish [11, 12]. Based on the genetic mechanism underlying the bolting characteristics of these plants, the identification method, the biochemical basis, and the molecular mechanism of each level were analyzed. Before and after bolting, the physiological and biochemical processes of plants undergo substantial changes, including carbohydrate, soluble protein, and free amino acid metabolism [13, 14].

However, there has been little analysis of bolting for “nonlow temperature vernalization”-type plants such as lettuce, and the molecular mechanism remains unclear. Lettuce (*Lactuca sativa L.*), as a cool-season vegetable, is susceptible to bolting when exposed to supra-optimal temperatures. The optimum growth temperature for lettuce is 15–20 °C, and temperatures greater than 30 °C promote early bolting, thus affecting the edibility [15]. Therefore, investigation of the molecular mechanism of bolting in lettuce caused by high temperature, inhibition of early bolting, and improvement of yield and quality are important. Currently, two genes, namely, *LsFT* and *LsSOC1,* are known to participate in the heat-promoted bolting process [16, 17]. The expression level of *LsFT* can be promoted by heat treatment, and knockdown of the expression of this gene in transgenic plants delayed bolting, and the plants failed to respond to high temperatures [16]. *LsSOC1* also functions as an activator of bolting during high-temperature treatment [17]. In addition, MADS-box genes and GAs can regulate bolting in lettuce [18]. Overexpression of *LsGA3ox1* may increase the GA1 content to promote early bolting in lettuce [19]. Transcriptomic analysis of lettuce heat treatment was performed and showed the upregulation of genes implicated in photosynthesis, oxidation-reduction and auxin activity [18]. However, the physiological and molecular basis of bolting initiation is poorly understood.

Proteins are the executors of physiological functions, so the study of protein structure and function can elucidate the changes in mechanism that occur under certain conditions. Therefore, it is necessary to assess the overall changes in intracellular proteins to reveal the mechanisms underlying plant physiological changes. The concept of the proteome was proposed in 1994 by Wilkins and refers to the total proteins expressed in a cell or tissue. Proteomics has been widely applied to explore the molecular mechanisms of plant disease resistance and stress resistance. Proteomic technology has been widely used to explore a variety of physiological and morphological changes associated with plant development and resistance to environmental factors such as the growth patterns of various stages of fruit development [20], disease resistance [21], heat resistance [22], cold resistance [23], and salt resistance [24, 25]. Currently, iTRAQ (isobaric tags for relative and absolute quantification) is the most popular technology for plant proteomics.

Here, comparative proteomics was used to increase our understanding of the mechanism of initiation of bolting in lettuce. We examined, for the first time, the global changes in the proteome following initiation of bolting using iTRAQ-based proteomic strategies coupled with liquid chromatography–tandem mass spectrometry (LC-MS/MS). The RT-qPCR, cytological observation and physiological analyses were used to verify the results. Our study is expected to identify the proteins or biological processes that participate in initiation of bolting caused by high temperature, revealing the molecular mechanism initiation of bolting in “nonlow temperature vernalization” type lettuce plants and providing a theoretical basis for the regulation of bolting and prevention of premature bolting.

## Results

### Effect of high temperature on bolting of lettuce

**Fig. 1:**
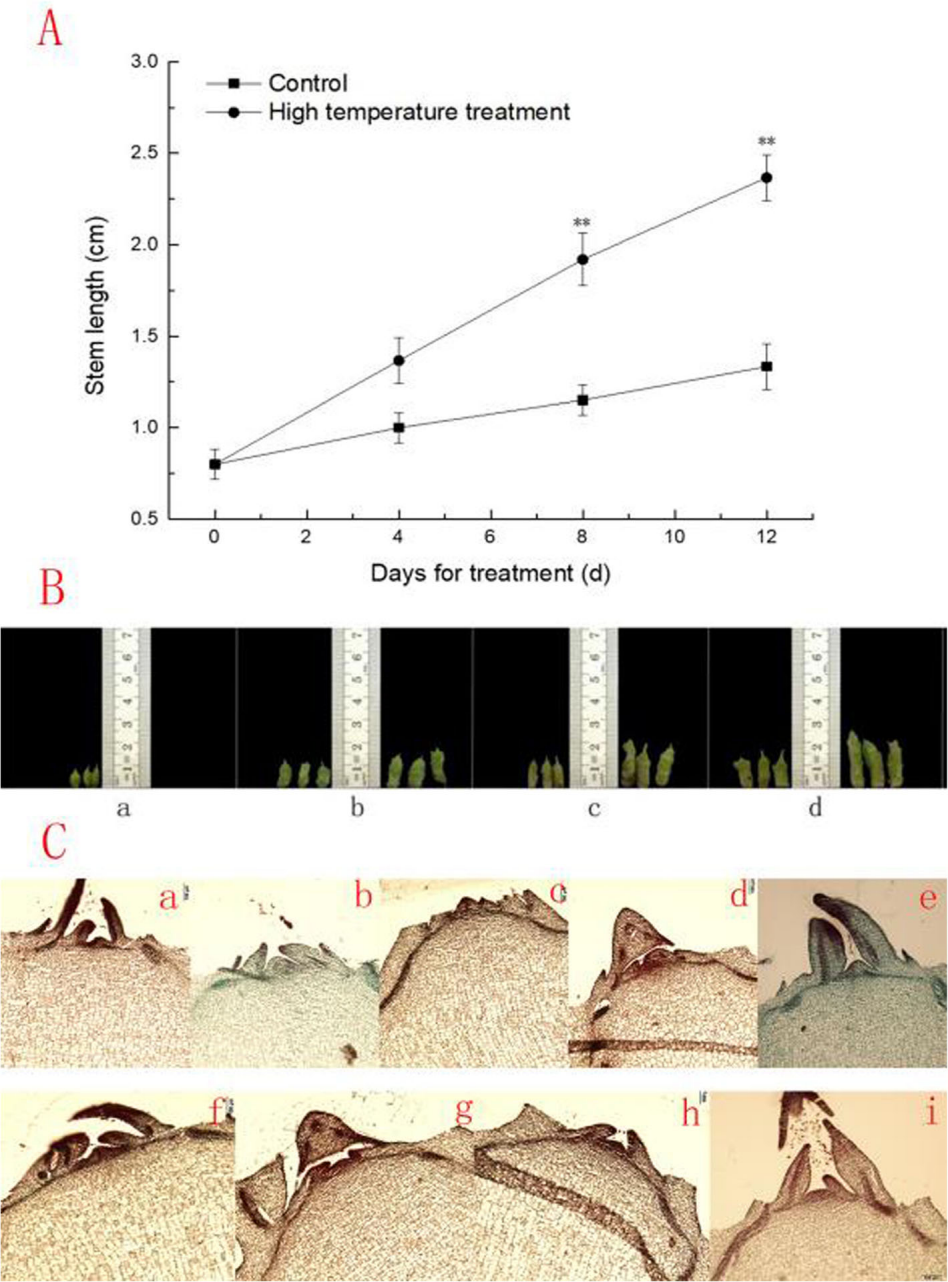
Change in stem height in lettuce after high-temperature treatment. **a** Changes in stem length under suitable temperature (control) and high temperature treatment. The data (mean **±** SD) are the means of three replicates with standard errors shown by vertical bars, *n* = 6. ^*^ and ^**^ indicate significant differences at *p* < 0.05 and *p* < 0.01 by *t*-test, respectively (**b**) The phenotypes of lettuce under different temperature treatments. a, b, c and d represent stem growth after different temperature treatments for 0, 4, 8 and 12 d. (control at left and high temperature treatment at right); (**c**) The progress of flower bud differentiation. a, b, c, d and e represent the morphology of flower buds under controlled temperature for 0, 2, 4, 6 and 8 d, and f, g, h and i represent the morphology of flower buds under high temperature conditions for 2, 4, 6 and 8 d

Bolting of lettuce can be induced by high temperatures [1]. To determine the stage initiation of bolting, we chose the easy bolting variety G-B30 as the test material and set two sets of temperature treatments: high temperature (33/25 °C) and control treatment (20/13 °C). We found that from the 8th day of high-temperature treatment, the lettuce stem elongation rate was significantly higher than the stem elongation rate observed for the control group (Fig. 1a and b). The stem of control group tip during the whole observation stay conical. The shoot tip growth point of the high temperature group was still remained conical up to the day 6. The growth point became larger and less pronounced on day 8, showing initiation of flower bud differentiation (Fig. 1c), which is consistent with our previous research results [26].

### Changes in sugar component levels in leaf lettuce during bolting induced by high temperature

**Fig. 2:**
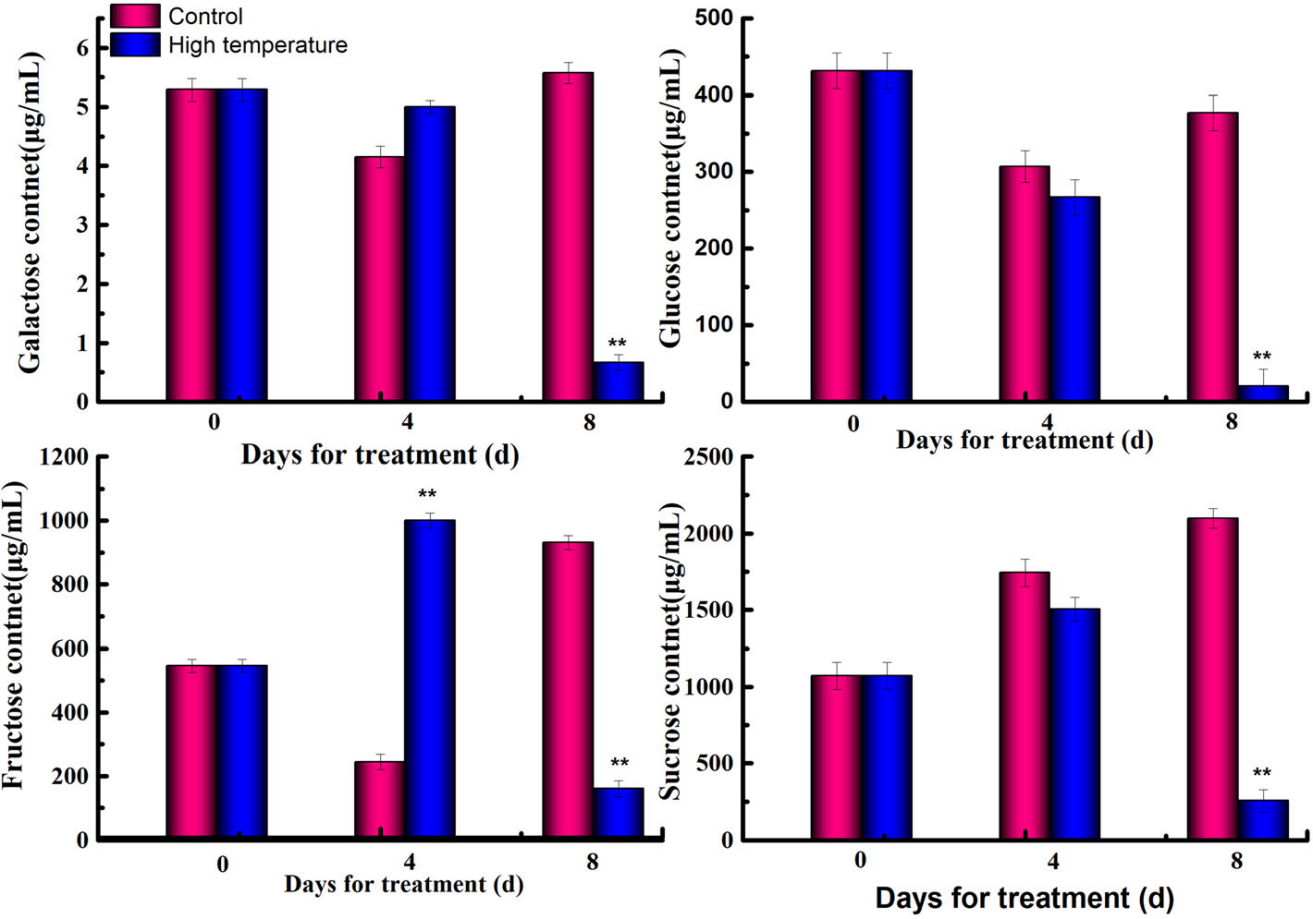
The contents of galactose, glucose, fructose and sucrose in lettuce in the control and after high-temperature treatment. The data (mean **±** SD) are the means of three replicates with standard errors shown by vertical bars, *n* = 3. ^*^ and ^**^ indicate significant differences at *p* < 0.05 and *p* < 0.01 respectively, by *t*-test

High temperature can lead to severe physiological responses such as changes in sugar component levels [27, 28]. Therefore, we tested the variations in the levels of four main sugar components, namely, galactose, glucose, fructose and sucrose, after high-temperature treatment. We found that the concentrations of galactose and glucose decreased gradually after high temperature treatmentx, while the fructose and sucrose levels increased first and then decreased after treatment. Among these sugar components, fructose production was rapidly induced by high temperature and peaked on the 4th day, exhibiting the opposite trend compared with the control group before the 8th day (Fig. 2). From the 4th day to the 8th day after treatment, the levels of all four sugars decreased rapidly and were lowest on the 8th day, while in the control group, all four sugars showed an increasing trend. The lowest concentrations of galactose, glucose, sucrose and fructose on the 8th day after treatment were 3.34, 101.20, 802.01 and 1278.72 μg/g, respectively. This result, together with the early bolting of shoots observed after 8 days of high-temperature treatment, showed that the 8th day was an important time point for lettuce bolting induced by high temperature.

### Identification of differentially abundant proteins using iTRAQ in lettuce during initiation of bolting induced by high temperature

**Fig. 3:**
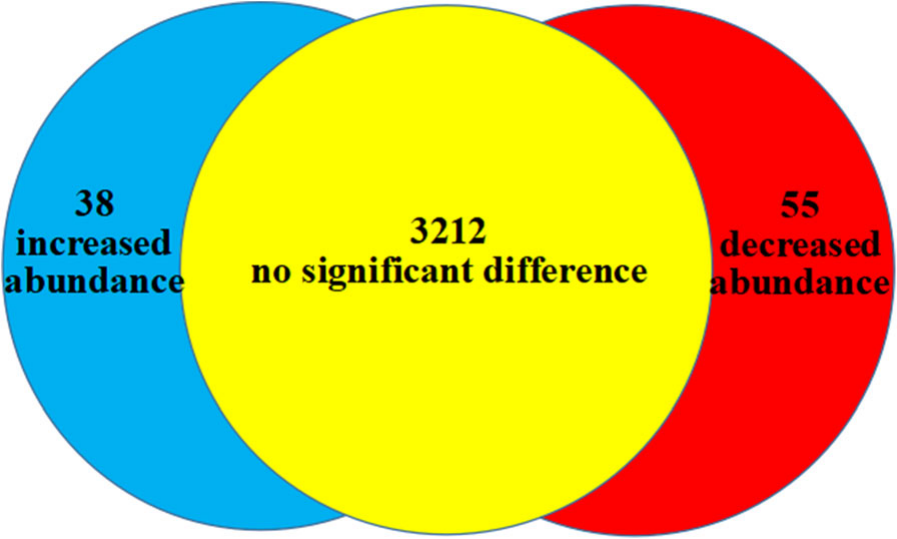
The distribution of differentially expressed proteins

Based on the above changes in phenotypes, cytological observations and physiological analyses after high-temperature treatment, we found that initiation of bolting occurred on the 8th day, so we analyzed the protein abundance between the control and treatment groups in this period using the iTRAQ-labeled proteomics approach. In total, 3305 proteins were identified (Table S1). The mass spectrometric proteomics data have been deposited in the ProteomeXchange Consortium (http://proteomecentral.proteomexchange.org) via the iProX partner repository with the dataset identifier PXD014464. Each high-confidence protein identification required at least one unique peptide, and quantification required at least two unique peptides. The abundances of 93 proteins changed significantly, and 38 of these proteins exhibited increased abundance (blue section in Fig. 3), while 55 proteins exhibited decreased abundance (red section in Fig. 3). Among these proteins, the upregulated proteins with the highest fold changes were aldehyde dehydrogenase family 2 member B4 (2.32), thaumatin-like protein (2.04) and rRNA 2′-O-methyltransferase fibrillarin-like protein (2.04). Interestingly, among the upregulated proteins, there were three heat shock protein-like proteins, namely, XP_023757207.1 (1.69), PLY74879.1 (1.50) and XP_023740399.1 (1.39). Detailed information on proteins with differential abundances is provided in Table S2. One GA-related protein 14-like protein was upregulated by high temperature (1.64). The most highly downregulated proteins were TsetseEP-like protein (0.32) and Calvin cycle protein CP12–3 (0.37). All peptide match information, including PSMs, PEP, Ionscore, expected value, charge, MH+ [Da], and ΔM [ppm], is provided in Table S3.

### Functional classification and metabolic pathways of differentially abundant proteins

**Fig. 4:**
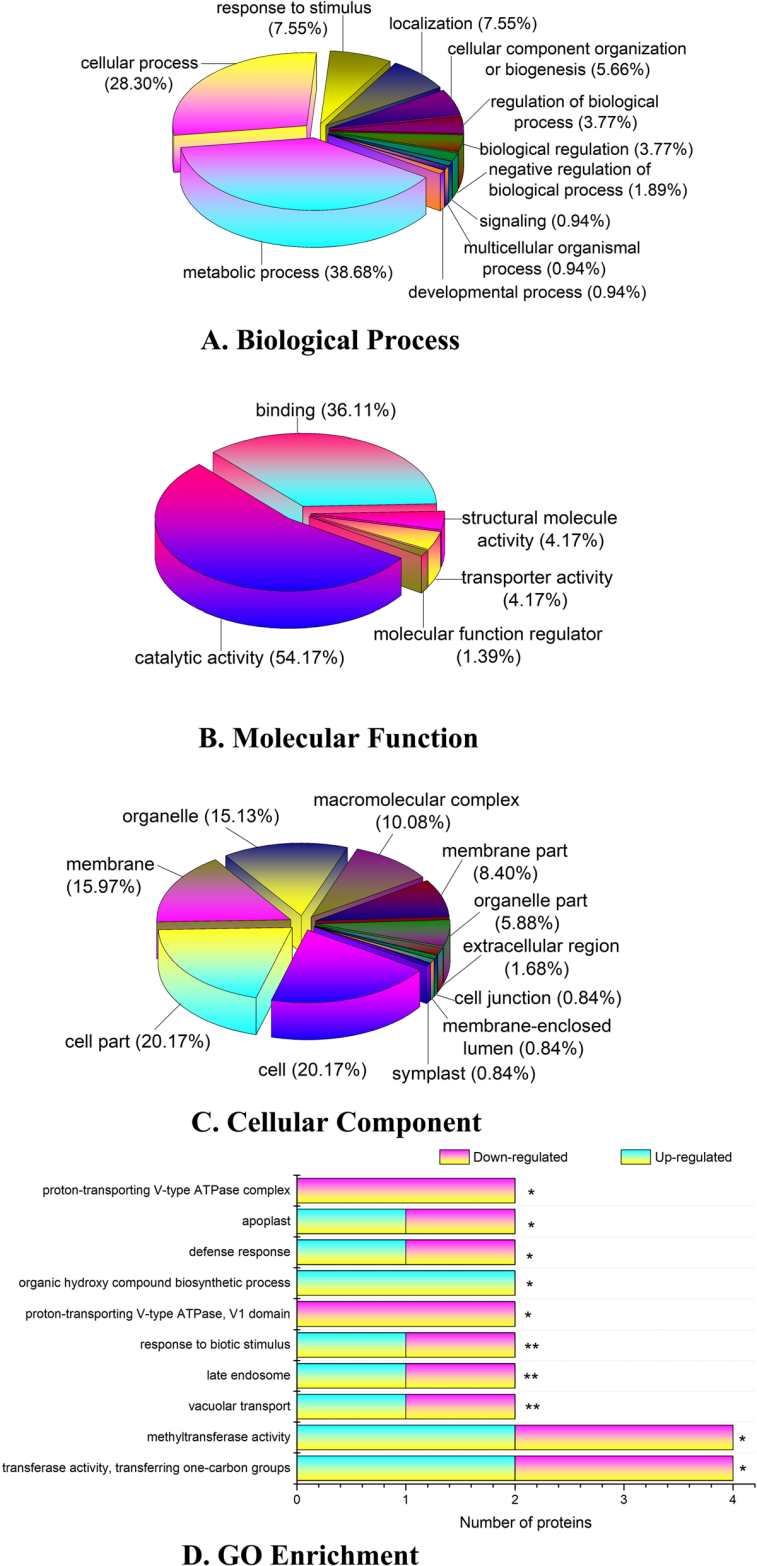
ClueGO and GO enrichment analysis of differentially expressed proteins. **a** Biological process; **b** molecular function; **c** Cellular component; **d** GO enrichment. ^*^ and ^**^ indicate significant differences at *p* < 0.05 and *p* < 0.01 by *t*-tests, respectively

To identify the proteins that regulate initiation of bolting induced by high temperature, we classified the differentially abundant proteins into 11 functional categories according to BLAST alignment, GO classification, and the literature [29]. GO annotation was performed using Trinotate through a BLAST search against SwissProt to identify the signal changes in BP, MF and CC after high-temperature treatment, and 603 GO terms were annotated. In the BP category, the proteins with differential abundance were annotated with the following terms: metabolic process (38.68%), cellular process (28.30%), response to stimulus (7.55%), localization (7.55%), cellular component organization or biogenesis (5.66%), and other terms (12.26%) (Fig. 4a). The five terms annotated in the MF category were catalytic activity (54.17%), binding (36.11%), structural molecule activity (4.17%), transporter activity (4.17%) and molecular function regulator (1.39%) (Fig. 4b). Similarly, the terms annotated in the CC category were cell (20.17%), cell part (20.17%), membrane (15.97%), organelle (15.13%) and macromolecular complex (10.08%) (Fig. 4c). Next, GO term enrichment analysis of the proteins identified with differential abundances showed that the main GO enrichment functions of the upregulated proteins were organic hydroxy compound biosynthetic process (2); transferase activity, transferring one-carbon groups (2); and methyltransferase activity (2). The main GO enrichment functions of the downregulated proteins were proton-transporting V-type ATPase complex (2); proton-transporting V-type ATPase, V1 domain (2); transferase activity, transferring one-carbon groups (2); and methyltransferase activity (2) (Fig. 4d).

**Fig. 5:**
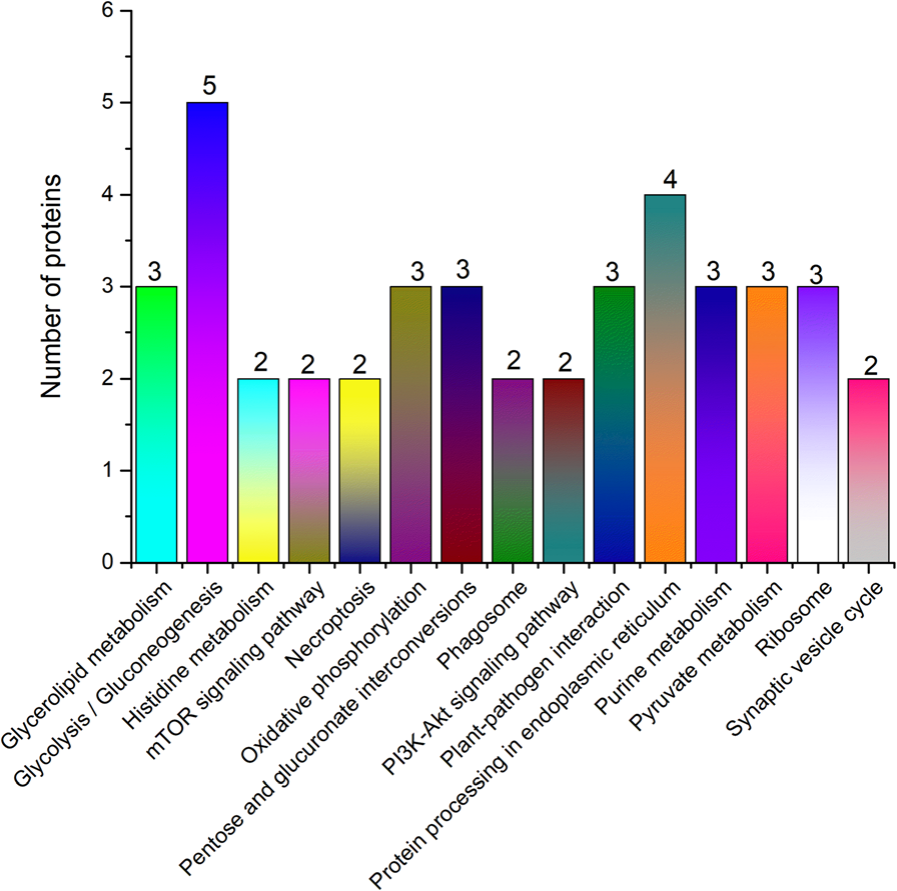
Metabolic analysis of KEGG pathway

To further identify the proteins with differential abundances that participate in major metabolic and signal transduction pathways, we analyzed the proteomic data based on the KEGG database [30]. A total of 107 KEGG signaling/metabolic pathways associated with 46 proteins were extracted. As shown in Fig. 5, the main metabolic pathways were glycolysis/gluconeogenesis (5), protein processing in the endoplasmic reticulum (4), glycerolipid metabolism (3), oxidative phosphorylation (3), pentose and glucuronate interconversions (3), plant-pathogen interaction (3), purine metabolism (3), pyruvate metabolism (3), ribosome (3), histidine metabolism (2), mTOR signaling pathway (2), necroptosis (2), phagosome (2), PI3K-Akt signaling pathway (2), and synaptic vesicle cycle (2).

The glycolysis process, oxidative phosphorylation, pyruvate metabolism, and pentose and glucuronate interconversions are involved in energy metabolism. In the glycolysis process, four proteins with increased abundances were identified, namely, pyruvate decarboxylase 1 like (PD1L), aldehyde dehydrogenase family 2 member B4 (ADF2MB4), pyruvate kinase 1 (PK1), and glyceraldehyde-3-phosphate dehydrogenase (G-3-PD), while NADPH-dependent aldo-keto reductase exhibited decreased abundance. In the oxidative phosphorylation pathway, the hypothetical protein LSAT_2X93901 was upregulated, while the V-type proton ATPase subunit C-like and V-type proton ATPase catalytic subunit A-like were downregulated. In pyruvate metabolism, three proteins, namely, the lactoylglutathione lyase GLX1, pyruvate kinase 1 (PK1), and aldehyde dehydrogenase family 2 member B4 (ADF2MB4), were upregulated. In the pentose and glucuronate interconversion pathway, NADP-dependent D-sorbitol-6-phosphate dehydrogenase-like exhibited increased abundance, while NADPH-dependent aldo-keto reductase and exopolygalacturonase-like exhibited decreased abundance.

In the ribosome, three ribosomal proteins, namely, 40S ribosomal protein S11–2 (40SRPS11–2), 40S ribosomal protein S5-like (40SRPS5l) and 60S ribosomal protein L32–1-like (60SRPL32–1 l), were differentially expressed. In protein processing in the endoplasmic reticulum, the proteins 17.5 kDa class I heat shock protein-like and heat shock protein 83-like were upregulated. These pathways play important roles in protein synthesis.

### The level of expression of genes that encode some identified proteins

**Fig. 6:**
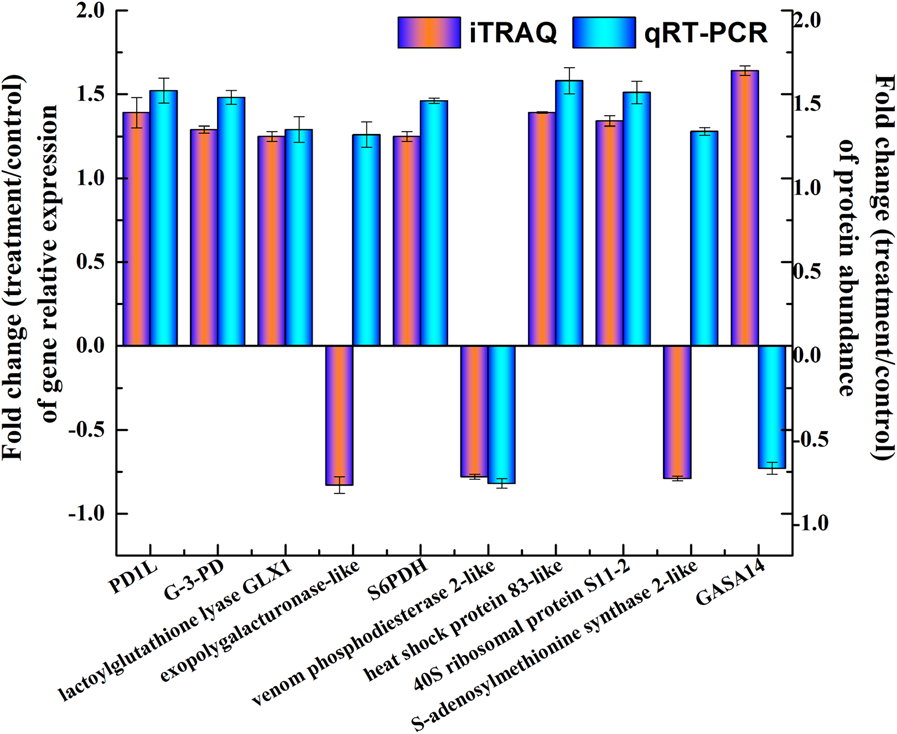
Correlation of mRNA level and protein abundance by iTRAQ. The fold-change of treatment/control at the transcript level using the RT-qPCR approach of 10 candidate genes involved in the identified differentially expressed proteins and the protein expression level by iTRAQ is shown in the figure. A positive number indicates upregulation, and a negative number indicates downregulation. Each histogram represents the mean value of three biological replicates, and the vertical bars indicate the standard error (*n* = 3). Definition of 10 candidate genes involved in the identified differentially expressed proteins: (1) PD1L, pyruvate decarboxylase 1 like; (2) G-3-PD, glyceraldehyde-3-phosphate dehydrogenase; (3) S6PDH, sorbitol-6-phosphate dehydrogenase; (4) GASA14, Gibberellin-regulated protein 14

We further confirmed the changes in protein abundance observed during bolting of lettuce by evaluating the changes in transcript levels and determined the relationship between the abundance of a protein and the level of the corresponding gene transcripts. Ten key node proteins were selected to measure the expression profiles for RT-qPCR analysis. Of the selected proteins, most of the genes showed change trends similar to the iTRAQ results. The mRNA expression trends for seven proteins, including PDIL, G-3-PD, lactoylglutathione lyase GLX1, sorbitol-6-phosphate dehydrogenase (S6PDH), venom phosphodiesterase 2-like, heat shock protein 83-like and 40S ribosomal protein S11–2, were consistent with the protein abundances (Fig. 6). However, the expression levels of three proteins (exopolygalacturonase-like, S-adenosylmethionine synthase 2-like, Gibberellin-regulated protein 14 (GASA14) were not consistent with the mRNA and protein levels. Possible reasons for this discrepancy may be posttranscriptional, translational, and posttranslational mechanisms or feedback loops between the processes of mRNA translation and protein degradation (Fig. 6).

## Discussion

### Differentially expressed proteins (DEPs) are involved in energy metabolism in the bolting process

During bolting, drastic changes occur in the cell and tissue, which is a process high energy demand. Most DEPs such as the glycolysis process, pyruvate metabolism, and pentose and glucuronate interconversion pathway were associated with energy metabolism. Additionally, most of these proteins exhibited increased abundances, implying that the accumulation of energy is preparatory for bolting, and these results are in line with the reproductive stage of the plant being a high energy consumption process [31]. The central role of glycolysis in plants is to provide energy in the form of ATP and to generate precursors such as fatty acids and amino acids for anabolism [32]. These findings are consistent with photosynthesis, carbon metabolism, and glycolysis/gluconeogenesis possibly played a crucial part in inducing the lettuce bolting [26]. Glycolysis play a crucial part in promoting development of bolting in plants. In this study, glyceraldehyde-3-phosphate dehydrogenase (G-3-PD), a key enzyme of glycolysis, was highly expressed after heat stress, and the transformation of glycolaldehyde-3p to glycolate-1,3p2 was accelerated. We also found that the glucose content was significantly lower than the glucose content of the control 8 days after high-temperature treatment. PK1 expression was increased, so the transformation of phoehoenolpyruvate to pyruvate was also promoted. With the increase in PK1 expression, the transformation from phoehoenolpyruvate to pyruvate was also promoted. With the increase of PD1L and ADF2MB4 expression and the decrease in alcohol dehydrogenase (AD) expression, pyruvate was finally transformed into acetaldehyde and acetate instead of ethanol. Among these proteins, G-3-PD is the key enzyme in the glycolytic metabolic pathway and is closely associated with energy generation [33]. G-3-PD can catalyze the conversion of glyceraldehyde 3-phosphate to 1,3-diphosphoglycerate in glycolysis, and in this oxidation process, energy is generated and stored as ATP. PK1 has crucial roles in the glycolysis pathway, where this enzyme catalyzes the final step of glycolysis. In particular, PK catalyzes the transfer of a phosphate group from phosphoenolpyruvate to ADP to yield one molecule of pyruvate and one ATP molecule. The glycolysis pathway facilitates the conversion of glucose to pyruvate, which can be used as a respiratory substrate [34]. Therefore, the amount of pyruvate that entered the TCA cycle increased, and thus, the amount of respiratory substrate increased, thereby accelerating the electron transport chain indirectly, which may have an effect on the process of bolting. In the present study, many documents indicate that high temperatures cause alterations in carbohydrate metabolism during the reproductive stage [35–37].

In addition, the protein lactoylglutathione lyase GLX1 in pyruvate metabolism was upregulated during bolting in response to high temperature. Lactoylglutathione lyase GLX1 catalyzes the conversion of hemimercaptal, formed from methylglyoxal and glutathione, to S-lactoylglutathione. This protein is involved in step 1 of the subpathway that synthesizes (R)-lactate from methylglyoxal [38]. The glyoxalase (GLX) system comprises two enzymes, namely, GLX1 and GLX2, and detoxifies MG. However, this system is poorly understood in the chloroplast compared with the cytosol. In *A. thaliana*, five GFP-fusion GLXs were present in the chloroplasts. Under high CO_2_ concentrations, at which increased photosynthesis promotes MG production, GLX1 and GLX2 activities in *A. thaliana* increased, and the expression of AtGLX1–2 and AtGLX2–5 was enhanced. Based on these findings and the phylogeny of GLX in oxygenic phototrophs, the GLX system scavenges MG produced in chloroplasts during photosynthesis [39]. These observations are consistent with the results that carbohydrate and energy metabolism is involved in floral bud development [40], which indicates that increased energy production via glycolysis and the pyruvate pathway can be redirected to initiation of bolting in lettuce.

### Fructose may participate in early bolting induced by high temperatures in lettuce

In all organisms, metabolic processes, including carbohydrate, amino acid, and lipid metabolism, are basic activities and are very sensitive to the environmental factors, such as temperature [41]. In the plant life cycle, bolting is the vital time node between vegetative growth to the reproductive stage, when nutrients are gradually redistributed to the reproductive organs. In lettuce, the SAM rapidly elongates into the floral stem at high temperatures (Fig. 1). According to our data, floral stem growth can be promoted by high-temperature treatment. We found that only fructose accumulated rapidly at high temperature on the 4th day, and the fructose content at high temperature was significantly higher than the fructose content in the control group (Fig. 2), while other sugars exhibited no difference in content on the 4th day of heating (Fig. 2). KEGG analysis showed that one protein, an aldehyde reductase, is involved in fructose and mannose metabolism, which is consistent with the above information (Fig. 2). Overall, we speculated that the expression of aldehyde reductase leads to fructose accumulation during the initiation of bolting induced by high temperature. Carbohydrates are not only important components of the cell but also the main source of energy. Therefore, fructose accumulation is also beneficial for increasing energy levels to meet the demand for bolting induced by high temperature.

Carbohydrates play an important role in plant adaptation to high temperature. The accumulation of sugar in plants can regulate the osmotic pressure of cells, enhance the water holding capacity of cells, and ultimately improve their own heat resistance. Researchers found that trehalose can enhance the tolerance of *Saccharomyces cerevisiae* cells to heat [42, 43]. Our previous studies also showed that high temperature induced bolting of lettuce was achieved by increasing photosynthesis, carbohydrate metabolism, etc. [26] In this study the content of fructose and sucrose was found to ireach the maximum value on the 4th day after high temperature treatment (Fig. 2). Biological information analysis shows that aldehyde reduction (AR) is involved in fructose and mannose metabolism, as well as galactose metabolism. With the increase in its expression, the transformation of D-sorbitol to α-D-glucose and D-galactose to galactitol was accelerated. Ectonucleotide pyrophosphatase/phosphodiesterase family member 1/3 (EPFM1/3) is involved in starch and sucrose metabolism. With the decrease in its expression, the conversion of UDP glucose to α-d-glucose-1p was inhibited, and the output of glycolysis products was accelerated. Galacturan 1,4-alpha-galacturonidase (G1,4-AG), alcohol dehydrogenase (NADP+) (AD,NADP+) and aldehyde reductase (AR) are involved in the pentose and gluconate conversion metabolic pathway. Along with the upregulation of AR, the downregulation of G1,4-AG and AD,NADP+ accelerates the mutual transformation between pentose and glucuronate. Carbohydrates play a crucial part in the plant response to high temperature according to all the results.

### DEPs are preferentially associated with protein synthesis and amino acid metabolism

Proteins are the basis of all life activities. Protein biosynthesis and amino acid metabolism must be involved in the substantial physiological and structural changes that occur in the cell and tissue during the bolting process. Ribosomes, as the sites of protein synthesis in cells, are one of the ribonucleoprotein particles present in cells and are composed of RNA and ribosomal protein. In ribosomes, amino acids are assembled into protein polypeptide chains based on the instructions encoded by the RNA. Early studies found that many ribosomal proteins are also essential for protein translation. Recently, researchers have found that the ribosome is involved in DNA repair, regulation of cell development and cell differentiation [44]. In plant ribosomal protein mutants, plant growth was inhibited [45]. For example, the 60S acidic ribosomal protein may affect flowering in plants by regulating the translation of flowering-related proteins [46]. In this study, we found that 3 ribosomal proteins, namely, 40SRPS11–2, 40SRPS5l and 60SRPL32–1 l, were included among the DEPs. Changes in 40S and 60S ribosomal proteins are observed in the flowering stage of winter wheat after heat stress treatment [47]. Selective protein degradation is necessary for plant growth and development and a variety of cellular regulatory processes. Proteases can hydrolyze proteins to amino acids and can be divided into endopeptidases and exopeptidases based on the hydrolysis site of the substrate. We identified five amino acid metabolism-related proteins involved in histidine metabolism; lysine degradation; beta-alanine metabolism; arginine and proline metabolism; valine, leucine and isoleucine degradation; glycine, serine and threonine metabolism; and cysteine and methionine metabolism. Among these proteins, the downregulated protein glycine hydroxymethyltransferase promotes glycine, serine and threonine metabolism by inhibiting the transformation of serine to glycine. S-adenosyl methionine synthetase promotes cysteine and methionine metabolism by inhibiting the conversion of L-methionine to S-adenosyl-L-methionine. Heat shock proteins (HSPs) or molecular chaperones are highly conserved protein families present in all studied organisms. Following cellular stress, the intracellular concentrations of HSPs generally increase several-fold. HSPs undergo ATP-driven conformational changes to stabilize unfolded proteins, unfold these proteins for translocation across membranes or mark these proteins for degradation. HSPs are broadly classified into several families according to molecular weights and functional properties. Extensive studies conducted over the past few decades have suggested that HSPs play a vital role in both normal cellular homeostasis and stress response. HSPs have been reported to interact with numerous substrates and are involved in many biological functions, such as cellular communication, immune response, protein transport, apoptosis, cell cycle regulation, gametogenesis and aging [48, 49]. Under the high temperature many proteins related to protein synthesis and amino acid metabolism were involved in physiological and morphological changes during the early bolting, according to all the data.

## Conclusion

According to phenotypic and cytological observations, we found that the initiation of bolting occurred 8 days after high-temperature treatment at 33 °C, with no bolting occurring at a normal temperature of 20 °C. To obtain further insights into the mechanism responsible for initiation of bolting, this study presents, for the first time, a comprehensive analysis of lettuce quantitative proteomics during initiation of bolting. During the initiation of bolting in lettuce, energy production appears to be enhanced or at least poised for enhancement. Energy metabolism can regulate gene expression (protein biosynthesis reinitiation). These cellular responses are responsible for the occurrence of flower bud differentiation and cell elongation in plants, thereby inducing bolting. The data set indicates that a complete understanding of the biochemical and physiological changes during initiation of bolting is possible for lettuce.

## Methods

### Plant materials and treatment

Leafy lettuce (*Lactuca sativa* L.) GB-30 (bolting resistant, numbered and conserved in our laboratory) was considered the experimental material. The seeds were sown in a sand/soil/peat (1:1:1 v/v) mixture and grown in the Beijing University of Agriculture Experimental Station in Beijing under standard greenhouse conditions (14 h light; 300–1300/mol/(m^2^ s); 20 ± 2 °C during the day; 13 ± 2 °C at night; 10 h dark; and 50–70% relative humidity). Pest control and water management were performed according to standard practices. When the sixth real leaves were formed, move the lettuce plants under the growing chamber in the following conditions: The temperature was 20 / 13 °C (day/night), the photoperiod was 14/10 h, and the relative humidity was 60% for 2 days of acclimation. Then, divide the plants into two groups. Keep the control group under standard greenhouse conditions where described above. The other group moved to another growth chamber, respectively for 33 / 25 °C high temperature treatment (day/night). Other environmental conditions remain unchanged.

The blossom buds were observed every two days by stereomicroscopy and paraffin-based methods [50] to monitor the progress of flower bud differentiation. A ruler was used to measure the stem length (in cm) of the control and treatment groups every 4 days. Simultaneously, collect the stem samples from control and treatment plant, freeze samples in liquid nitrogen, and store them at − 80 °Crefrigerator for further physiological analysis. On day 8, collect stem samples from the control and treatment plants, freeze samples in liquid nitrogen, and store them at − 80 °C refrigerator for further proteome analysis.

### Measurement of sugar components

Approximately 0.2 g samples were ground in 10 mL 80% (v/v) ethanol in a tube, and then the tube was placed in a boiling water bath for 1 h, cooled, and centrifuged at 1000 g for 10 mins. The pellet was extracted two additional times with 10 mL 80% (v/v) ethanol. The supernatants from each extraction were combined and evaporated to dryness in a boiling water bath. The samples were resolubilized in 0.5 mL distilled water and filtered through an acetate filter (0.45-μm pore size, Nalgene, Thermo Fisher Scientific, Waltham, MA).

The contents of galactose, glucose, fructose, and sucrose were determined using high-performance liquid chromatography (HPLC) [51]. The system included a Waters 6000A pump (Millipore, Waters Chromatography Division, Milford, MA), an Inertsil NH2 column (250 mm × 4.6 mm, 5 μm, Dikma Company, Forest Lake, CA) and a Waters 2410 refractive index detector connected to a strip chart recorder. Distilled water, at a flow rate of 10 mL/min, was used as the solvent of the 70% (v/v) acetonitrile. The column temperature was maintained at 35 °C and was preceded by a Waters Bondapak C18/Corasil guard and a set of anion and cation cartridges (deashing guards, Bio-Rad Laboratories, Richmond, CA). All guards were operated at an ambient temperature of 25 °C, and 20 μL samples were injected. Galactose, glucose, fructose, and sucrose were identified and quantified from the retention times and the peak heights of stachyose and raffinose standards. All chemicals were of chromatographic grade in purity. Standards of stachyose and raffinose were purchased from Sigma (St. Louis City MO).

### Protein extraction

Extract the samples using the trichloroacetic acid (TCA)/acetone method as previously described with some modifications. Ground each sample into a fine powder in liquid nitrogen at about 2.5 g. Resuspend the powder in 30 mL of 10% (w/v) TCA/acetone (65 mM dithiothreitol (DTT) in a 50-mL tube. For precipitation, store the mixture overnight at least in − 20 °C refrigerator. Centrifuge the mixture at 4 °C at 10,000 rpm for 30 mins, and discard the supernatant. Then add 40 mL precooled acetone and centrifuge at 7000 rpm for 15 mins. Discard supernatant and wash the pellets with acetone for 3 times. Add 200 μL of lysis buffer (SDT buffer (4% (v/v) SDS, 100 mM Tris-HCl, 1 mM DTT, pH 7.6)) to the precipitate and ultrasonic treat for 30 mins, then place the mixture on ice for 20 mins. Centrifuge at 12,000 rpm at 4 °C for 10 mins and extract the supernatant. Afterwards, vacuum drying the precipitation. Quantitative the supernatant of total protein with BCA Protein Assay Kit (Bio-Rad, Hercules, CA, USA).

### Protein digestion and iTRAQ labeling

According to the Wi’sniewski and colleagues [52] described FASP application for protein digestion, and according to the manufacturer’s instructions (Applied Biosystems, Foster City, CA, USA) using the 8-plex iTRAQ reagent are labeled by the peptide mixture. In brief, incorporate each 200-μg protein sample into 30 μL of SDT buffer (4% (v/v) SDS, 100 mM DTT, 150 mM Tris-HCl, pH 8.0). Use UA buffer (8 M urea, 150 mM Tris-HCl, pH 8.0) and repeated ultrafiltration (Microcon units, 30 kDa) to remove the detergent, DTT, and other low-molecular-mass components. Then, add 100 μL of 0.05 M iodoacetamide (C_2_H_4_INO) in UA buffer to block reduced L-Cysteine residues, and incubate the samples in darkness for 20 mins. Flush the filter with 100 μL UA buffer solution 3 times and 100 μL DS buffer solution (50 mM triethylammonium bicarbonate at pH 8.5) 2 times. Finally, add 2 μg of trypsin (Promega, Madison, WI, USA) into 40 μL of DS buffer to digest the protein suspensions overnight at 37 °C, and collect the resulting peptides as filtrate. In the solution of 0.1% (g/L) concentration, the UV spectral density at 280 nm was determined by extinction coefficient of 1.1, and the peptide content was calculated according to the frequencies of tryptophan and tyrosine in vertebrate proteins. Dissolve each iTRAQ reagent into 70 μL of ethanol and add them to the relevant peptide mixture for labelling. The experiment was conducted in three separate biological duplications, each containing three plant pools. Mark the three separate biological duplications of the control as (CK1)-113, (CK2)-114, and (CK3)-115; mark the three separate biological duplications of the treatment as (H1)-116, (H2)-117, and (H3)-118.

### Separation of peptides by strong cation exchange (SCX) chromatography

The iTRAQ-labeled peptides by strong cation exchange (SCX) chromatography using AKTA filter system (GE Healthcare, Chicago, IL, USA) for separation. Reconstitute and acidify the dried peptide mixture with 2 mL of buffer A (10 mM KH_2_PO_4_ in 25% (v/v) acetonitrile (ACN), pH 2.7) and loaded into a PolySULFOETHYL 4.6 × 100 mm column (5 μm, 200 Å, PolyLC Inc., Columbia, MD, USA). Use of buffer B (500 mM KCl, 10 mM KH2PO4 in 25% (v/v) ACN, pH 2.7) to 1 mL/min flow elution peptide, gradient is as follows: 0–8% buffer B (500 mM KCl, 10 mM KH_2_PO_4_ in 25% ACN, pH 3.0) for 22 mins, 8–52% buffer B for 22–47 min, 52–100% buffer B for 47–50 min, 100% buffer B for 50–58 min. Then, after 58 mins, buffer B was reset to 0%. Absorbance was measured at 214 nm, elution was monitored, and fractions were collected per minute. Combine the collected moieties into 15 fractions and desalt them on C18 cartridges (Empore™ SPE C18 cartridges (standard density), bed inner diameter (I. D.) 7 mm, volume 3 mL; Sigma, St. Louis, MO, USA). Each component by vacuum centrifugal concentration, restructuring in the 40 mu L 0.1% (v/v) acetic acid. Store all samples at − 80 °C refrigerator until LC-MS/MS analysis.

### Analysis of liquid chromatography (LC)-electrospray ionization (ESI) tandem mass spectrometry (MS/MS)

Performing Experiments on a Q-Exactive mass spectrometer with the addition of an Easy nLC (Proxeon Biosystems, now Thermo Fisher Scientific). Nano-LC-MS /MS analysis was performed by injecting 10 μl of each component. Load the peptide mixture (1–2 μg) onto a C18 reversed-phase column (Thermo Scientific Easy Column, 10 cm length, 75 μm I.D., 3 μm resin) in buffer A (0.1% (v/v) formic acid) and separate the mixture with a linear-gradient of buffer B (80% (v/v) acetonitrile (C_2_H_3_N) and 0.1% (v/v) methanoic acid) at a flow rate of 250 nL / min, control it by IntelliFlow technology, for 140 mins. MS data were obtained using a data-dependent top 10 method, which dynamically select the most richest precursor ions from the survey scan (300–1800 m/z) for HCD fragmentation. Determine the target value of automatic gain control (pAGC) is based on prediction, and the dynamic elimination time is 60 s. The resolution of the survey scans was set to 70,000 at m/z 200, and the resolution of the HCD spectra was set to 17,500 at m/z 200. The normalized collision energy was 30 eV, and the underfill ratio was defined as 0.1%, which specifies the minimum percentage of the target value might be reached at the maximum fill time. The instrument operates in peptide recognition mode.

### Database search and protein quantification

MS/MS mass spectra were searched using the MASCOT engine (Matrix Science, London, UK; version 2.2) flushbonading into Proteome Discoverer 1.3 (Thermo Electron, San Jose, CA, USA) against Lactuca.Unigene.pep.fasta (a lettuce protein database which has 53,584 entries in total, was translated from the transcriptome and created by our research group). The material here was G-B30, which is Consistent with the experimental study of varieties). Use the following settings for protein identification: peptide mass tolerance = 20 ppm; MS/MS tolerance = 0.1 Da; enzyme = trypsin; max missed cleavage = 2; fixed modification: carbamidomethyl (C), iTRAQ 8-plex (K), iTRAQ 8-plex (N-term); variable modification: oxidation (M), iTRAQ 8-plex (Y). The false discovery rate (FDR) of protein identification was ≤0.01. At least one kind of unique peptides involved every highly credible protein identification.

Protein relative quantification was based on the reporting ion signal intensity, it reflected the relative abundance of the peptide. As described above, the protein ratios (fold change) of different test groups (high temperature treatment/control) were obtained by using the ratios of reported ions labelled with different isotopes. For differentially expressed proteins (DEPs), one can use a protein containing at least two unique spectra, and only these unique spectra, for quantification. Only fold changes ≥1.20 or ≤ 0.83 (the ratios with *p*-values < 0.05 and expected cutoff values < 0.05 with 95% confidence) were considered significant. Median intensities were used for normalization, and outliers were removed automatically (the quantitative protein ratio was normalized by the median ratio in MASCOT).

### Bioinformatic analysis of proteins

Function classification analysis was carried out by using Blast2GO software (http://www.geneontology.org) [53]. The KEGG Orthology (KO) data was retrieved using an online Kyoto Encyclopedia of Genes and Genomes (KEGG) database (http://www.genome.jp/kegg/) and then mapped to pathways in the KEGG database [54]. The corresponding KEGG pathways were extracted. Perform enrichment analysis for further explore the effects of different abundance proteins on the physiological process of cells and to determine the internal associations between different abundance proteins. Gene Ontology (GO) enrichment analysis was performed for three ontologies (biological process (BP), molecular function (MF), and cellular component (CC)). The derived p-values were further adjusted using the multi-test Benjamini-Hochberg correction, and only functional categories and pathways with p-values < 0.05 were considered significant.

### Total RNA extraction and RT-qPCR analysis

Transcript levels of genes associated with DEPs were determined using real-time quantitative polymerase chain reaction (RT-qPCR). For total RNA extraction, extract stems using an RNA Rapid Extraction Kit (Aidlab Biotech, Beijing, China) according to the operation manual. Use the Reverse Aid First Strand cDNA Synthesis Kit (TaKaRa Biotech, Beijing, China) to reverse-transcribe RNA to cDNA. The process was as follows: Mix RNA (2 μg) with 1 μL Oligo d (T) 18 (0.5 μg/μL), 2 × TS Reaction Mix (10 μL) and TransScript RT/RI Enzyme Mix (1 μL) with an extra 20 μL of RNase-free Water. Mix the mixture gently and incubate at 42 °C for 15 mins. Terminate the reaction by incubation at 85 °C for 5 s, and store the cDNAs of the product at − 20 °C. Use the cDNA samples as a template, and then mix them with 200 nmol primer and SYBR Green PCR Real Master Mix (TakaRa, Kusatsu, Japan) for real-time PCR analysis using Bio-Rad CFX 96 real-time PCR instruments and CFX manager software ver 3.0 (Bio-Rad Laboratories, California, USA). The PCR temperature procedure is as follows: 95 °C of predegeneration for 3 mins, 40 cycles of denaturation at 95 °C for 20 s, annealing at 59 °C for 20 s, and extension at 72 °C for 20 s. Use the 18S sequence as an internal standard for standardization.

### Statistical analysis

All tests were performed in triplicate. For stem length measurements, each biological replicate came from 6 samples of 6 plants. 5 samples per biological replicate from 6 plants were observed during flower bud differentiation. For physiological and proteomic analysis, 3 different stems were combined into a single biological sample and performed 3 times to produce 3 independent biological replicates (of three pooled stems). The data provided means ± SDs of three replications and were statistically analyzed using analysis of variance (ANOVA) with SPSS 10.0 (International Business Machines, Corporation (IBM), Chicago, IL, USA). To identify significant differences among groups (*p* < 0.05, *p* < 0.01) Tukey’s test was used. Figures representing the physiological parameters were generated automatically with both Origin Pro 8.0 SR4 (Origin Lab, Northampton, MA, USA) and Microsoft Office PowerPoint 2007.

## Supplementary Information

**Additional file 1: Table S1.** All proteins identified using iTRAQ in lettuce stems during bolting initiation induced by high temperature.

**Additional file 2: Table S2.** The differentially expressed proteins in lettuce stem during bolting initiation induced by high temperature.

**Additional file 3: Table S3.** All peptides identified using iTRAQ in lettuce stems during bolting initiation induced by high temperature.

## Data Availability

The following are available online at www.mdpi.com/link. Table S1: All proteins identified using iTRAQ in lettuce stems during bolting initiation induced by high temperature, Table S2: The differentially expressed proteins in lettuce stem during bolting initiation induced by high temperature, Table S3: All Peptides Identified Using iTRAQ in Lettuce stems during bolting initiation induced by high temperature.

## Abbreviations

SAM: Shoot apical meristem
IM: Inflorescence meristem
GA: Gibberellin
FT: FLOWERING LOCUS T
SOC1: SUPPRESSOR OF OVEREXPRESSION OF CONSTANS1
LFY: LEAFY
LC-MS/MS: Liquid chromatography–tandem mass spectrometry
PD1L: Pyruvate decarboxylase 1 like
ADF2MB4: Aldehyde dehydrogenase family 2 member B4
PK1: Pyruvate kinase 1
G-3-PD: Glyceraldehyde-3-phosphate dehydrogenase
PK1: Pyruvate kinase 1
ADF2MB4: Aldehyde dehydrogenase family 2 member B4
40SRPS11–2: 40S ribosomal protein S11–2
40SRPS5l: 40S ribosomal protein S5-like
60SRPL32–11: 60S ribosomal protein L32–1-like
S6PDH: Sorbitol-6-phosphate dehydrogenase
GASA14: Gibberellin-regulated protein 14
DEPs: Differentially Expressed Proteins
G-3-PD: Glyceraldehyde-3-phosphate dehydrogenase
AD: Alcohol dehydrogenase
GLX: Glyoxalase
AR: Aldehyde reduction
EPFM1/3: Ectonucleotide pyrophosphatase/phosphodiesterase family member 1/3
G1,4-AG: Galacturan 1,4-alpha-galacturonidase
AD,NADP+: Alcohol dehydrogenase
HSPs: Heat shock proteins
HPLC: High-performance liquid chromatography
TCA: Trichloroacetic acid
DTT: Dithiothreitol
SCX: Strong cation exchange
ACN: Acetonitrile
pAGC: Predictive automatic gain control
DEPs: Differentially expressed proteins
KEGG: Kyoto Encyclopedia of Genes and Genomes
KO: KEGG Orthology
GO: Gene Ontology
BP: Biological process
MF: Molecular function
CC: Cellular component
ANOVA: Analysis of variance
IBM: International Business Machines, Corporation
